# Encapsuled Placental Tumor Extracted from the Uterus Five Weeks after Parturition

**Published:** 1879-08-20

**Authors:** A. G. Hobbs


### *AN ENCAPSULED PLACENTAL TUMOR EXTRACTED FROM THE UTERUS FIVE WEEKS AFTER PARTURITION.*

A.

BY H. G. HOBBS, M.D.

I was called in March, 1879, to see Mrs. F—— in great haste, her husband stating that she was flooding. Upon inquiry, after my arrival at her bed side, I found she was five months *enceinte* in her eighth gestation. She had lifted a log in the morning, and since that time there had been a continual but slow oozing of blood, but no pains.

Upon examination I found the os dilated to the size of a silver half-dollar, but so high that I could hardly reach it with my index finger. I could distinctly feel, just within the right margin of the os, the doughy placenta, thus confirming my fears that it was a case of partial placenta prævia. In view of the entire absence of all uterine pains, and also of the small extent of the laceration of the placental margin, I gave it as my opinion that with absolute rest, together with astringent injections and opium with plumbi acet. internally, the danger of an abortion might be bridged over. I might say, by the way, that I gave fl. ext. viburnum prun.

I heard nothing more from the case till just a month after my first visit I was sent for in haste, her husband stating that the flooding had returned. I found her this time in about the same condition that I had at my previous visit. Upon inquiry I found she had been doing light household duties during the previous three weeks and was feeling well, except considerably weakened; the flooding, she said, had come on quite suddenly and without any pain.

I simply repeated the previous prescription, and at my visit next day the oozing had ceased. She was anemic and weak from loss of blood, so I put her upon a tonic of quinine and iron. There seemed to be a gradual improvement till just one month after her last attack, when I was sent for in haste again. I was absent, and in consequence of the emergency of the case, her husband went for another physician, and left word for me to come immediately upon my return. When I arrived at her bed three or four hours after the beginning of her attack, I found the doctor had delivered her of a 2 pound child. She was almost moribund from loss of blood; radial pulse not perceptible; opened her eyes occasionally with a staring look; pupils dilated; respiration sighing; face palid and suffused with cold perspiration. The doctor told me the placenta had been delivered, and I made no examination. By the cautious use of stimulants and ergot, together with cold applications to the vulva and lower bowels, and hot ones to the extremities, the hemorrhage ceased and the color returned to her face. When I saw her two days afterwards she was doing very well, with the exception of an occasional oozing of arterial blood. I instituted an examination but could find nothing abnormal within the reach of my finger. I left her upon tonic and an antiseptic injection.

I saw her again five weeks after the birth of her child; she had improved but slowly, with still an occasional oozing of blood. Upon examination this time I discovered what seemed to be an encapsuled bipedunculated or cauliflower-shaped tumor attached about an inch within the margin of the os, which was still dilated to the size of a silver dollar. I was not provided with a speculum or instruments suitable for its extraction; was consequently compelled to delay the operation till the next day. I inquired of the doctor if the case had been one of placenta prævia; he said it had not. I then asked him if the placenta had been delivered entire; he told me he thought it had. I possessed no *ecraseur* nor any other instrument suitable for extracting the tumor, but necessity is said to be the mother of invention, and I

improvised a rude *ecraseur* of small wire, and with this little instrument I performed the operation through a speculum.

The little instrument was rudely made by doubling a small wire about two feet in length upon itself; a small loop made in one end of the wire and the other left straight and run through it. By pushing upon the looped end and pulling upon the straight end the fold which had passed through the speculum enlarged, and grasped the tumor; then by a *vice versa* movement the loop decreased and together with traction cut its way through the pedicle. The tumor proved to be a placental mass somewhat larger than a goose's egg encapsuled with a thin membrane.

The stump bled profusely, and having no other styptic at hand I injected dilute tinc. ferri directly against the stump. The speculum was immediately filled with clotted blood, which I left in situ to stop the hemorrhage. I remained with her a couple of hours, then removed the clots and speculum when but a little oozing followed. I introduced a carbolized tampon and left her.

I removed the tampon the next morning and washed out the uterus with carbolized water. She convalesced rapidly and is now in good health. The child lived 36 hours.

My conclusions are that the doctor did not recognize the partial placental presentation probably in consequence of the small size of the child's head to which the placenta gave but little resistance, and he was careless in delivering the placenta or used too much traction upon the cord, and ruptured it at its point of greatest adhesion to the uterine walls.

Septicemia was evidentiy prevented by the astringent and antiseptic injections and perhaps these were the causes of the placental mass becoming encapsuled.

